# A practical overview of molecular replacement: *Clostridioides difficile* PilA1, a difficult case study

**DOI:** 10.1107/S2059798320000467

**Published:** 2020-02-26

**Authors:** Adam D. Crawshaw, Arnaud Baslé, Paula S. Salgado

**Affiliations:** a Newcastle University Biosciences Institute, Faculty of Medical Sciences, Newcastle University, Newcastle upon Tyne, England

**Keywords:** molecular replacement, *CCP*4*i*2 pipelines, *ARCIMBOLDO*, type IV pili

## Abstract

Many biologists are now routinely seeking to determine the three-dimensional structures of their proteins of choice, illustrating the importance of this knowledge, but also of the simplification and streamlining of structure-determination processes. Despite the fact that most software packages offer simple pipelines, for the non-expert navigating the outputs and understanding the key aspects can be daunting. Here, the structure determination of the type IV pili (TFP) protein PilA1 from *Clostridioides difficile* is used to illustrate the different steps involved, the key decision criteria and important considerations when using the most common pipelines and software. Molecular-replacement pipelines within *CCP*4*i*2 are presented to illustrate the more commonly used processes. Previous knowledge of the biology and structure of TFP pilins, particularly the presence of a long, N-terminal α-helix required for pilus formation, allowed informed decisions to be made during the structure-determination strategy. The PilA1 structure was finally successfully determined using *ARCIMBOLDO* and the *ab initio* MR strategy used is described.

## Abstract 2

A practical perspective on molecular-replacement (MR) structure-determination pipelines is presented, using PilA1 from *Clostridioides difficile* as an example of a difficult case. A manual approach informed by the biology of the system under study is described, together with *ab initio* MR structure determination.

## 1. Introduction   

**Figure 1:**
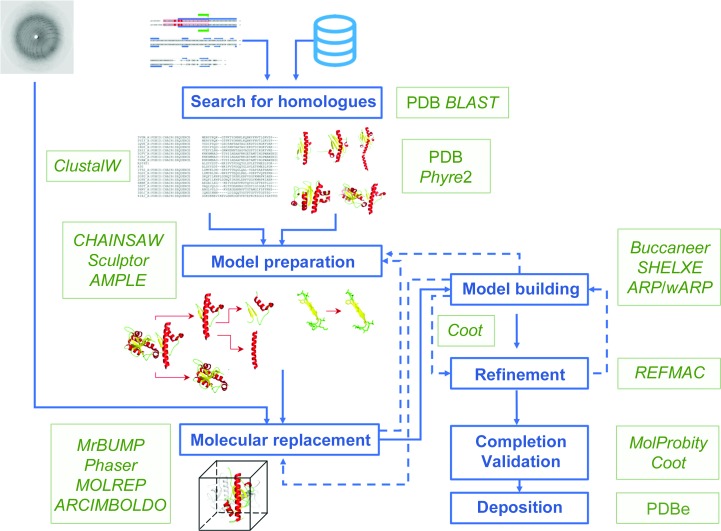
MR pipelines. Overall schematic of molecular-replacement pipelines and the currently available and frequently used programs. A particular focus is given to the pipelines and programs available within the *CCP*4*i*2 suite.

Once complete diffraction data at an appropriate resolution (usually at least around 3–3.5 Å) have been recorded from a protein crystal sample, the next challenge is to solve the phase problem (Taylor, 2003 ▸). Phases are required to calculate an electron-density map, from which a model of the structure is built. These are missing from diffraction data experiments and can be experimentally derived using techniques such as single/multiple anomalous dispersion (SAD/MAD) or single/multiple isomorphous replacement (SIRAS/MIRAS) (Taylor, 2010 ▸; Wang *et al.*, 2014 ▸). Alternatively, phases can be calculated using similar structures that have already been determined, which is known as molecular replacement (MR). Here, we review an outline of a number of different strategies (Fig. 1 ▸) starting with a single sweep of X-ray diffraction data to calculate an electron-density map using molecular replacement. We use the structure determination of the major pilin in type IV pili (TFP), PilA1, from *Clostridioides difficile* as a working example.

Molecular replacement can be used to determine phases for new structures using diffraction data of reasonable quality (*R* factors below 50%). While high-resolution data are preferable, it is the low-resolution information that is most important for molecular replacement, and there are a number of examples in the PDB of structures phased by molecular replacement where the data do not extend beyond 10 Å resolution (Evans & McCoy, 2008 ▸). MR does not require considerations such as the pre-soaking of crystals with heavy atoms or production, purification and crystallization that incorporates an anomalously scattering atom such as selenium, or determining the specific X-ray wavelength at which the data need to be recorded for experimental phasing (Garman & Murray, 2003 ▸). Although techniques such as sulfur SAD (S-SAD) enable experimental phases to be calculated from a ‘native crystal’, careful design of the diffraction experiment is required to be able to exploit the very weak anomalous differences, in addition to the difficulties in collecting data at the long wavelengths needed (Basu *et al.*, 2019 ▸; Bent *et al.*, 2016 ▸). Recent advances in both algorithms and instrumentation, including long-wavelength beamlines such as I23 at Diamond Light Source (Aurelius *et al.*, 2017 ▸), have rendered S-SAD an increasingly popular structure-determination method.

In any case, where crystal growth or supply are difficult, being able to determine the structure ‘natively’ can be particularly advantageous. Molecular replacement is also a powerful way to identify ligands, whether these are potential drugs, substrates or other compounds. After soaking or co-crystallizing experiments using different ligands, data sets recorded from multiple crystals of the same protein species in different conditions are used for phase calculation in search of a positive difference density representing a ligand in a potential binding site (Krojer *et al.*, 2017 ▸; Silvestri *et al.*, 2018 ▸).

Over the years, a number of different programs have been developed, some of which involve a more hands-on approach requiring suitable search models to be identified and prepared, such as *Phaser* (McCoy *et al.*, 2007 ▸, 2018 ▸) and *MOLREP* (Vagin & Teplyakov, 2010 ▸). Other programs such as *AMPLE* and *MrBUMP* provide a more complete pipeline, requiring only scaled reflections, the target sequence and, in the latter case, a small number of fragments provided by another server, *Rosetta* (Bibby *et al.*, 2012 ▸; Rigden *et al.*, 2008 ▸). Another approach is to use *BALBES*, a fully automated pipeline that uses a reorganized protein structure database derived from the PDB in order to include multimeric and domain organization models (Long *et al.*, 2008 ▸). Although these and other MR pipelines are available in different software packages such as *CCP*4*i*2 (Potterton *et al.*, 2018 ▸) and *Phenix* (Liebschner *et al.*, 2019 ▸), here we focus on *CCP*4*i*2.

More recently, it has increasingly become possible to determine phases using methods that do not use structural homologues of the full protein or domains. *ARCIMBOLDO* uses a fragment-based method and harnesses the predicted secondary structure of the target sequence to place fragments as search models (Millán *et al.*, 2015 ▸), in combination with powerful density-modification algorithms such as those implemented in *SHELXE* (Sheldrick, 2010 ▸), to provide an initial trace model (Usón & Sheldrick, 2018 ▸). This *ab initio* method was initially developed and based on the presence of α-helical structures in the target structure and reflections in the X-ray data that extend to approximately 2 Å resolution. More recent versions of *ARCIMBOLDO* now include the determination of β-strand structures (Fedosyuk *et al.*, 2016 ▸; Sammito *et al.*, 2013 ▸) and have also proven successful in solving the phase problem for data to 2.6 Å resolution.

The working example described here is the PilA1 protein from *C. difficile*. PilA1 is the major pilin of the type IV pili (TFP) system in this clinically important Gram-positive bacterium (Maldarelli, Matz *et al.*, 2016 ▸). *C. difficile* is an opportunistic pathogen, particularly affecting individuals with a compromised gut microbiome, often owing to treatment with antibiotics (Huang *et al.*, 2009 ▸). Pili are long, proteinaceous filaments which protrude from the surface of bacteria. They are virulence factors which enable host colonization, either by mediating interactions with host tissue, enabling direct binding of the bacteria to the host and/or by mediating inter-bacterial interactions, enabling bacterial aggregation/biofilm formation (Manetti *et al.*, 2007 ▸). TFP are the most widespread type of pili, and are uniquely found in both Gram-positive and Gram-negative species (Melville & Craig, 2013 ▸). In *C. difficile* and related Clostridia, they have been implicated in twitching ability and biofilm formation, both of which are important pathogenicity factors (Maldarelli, Piepenbrink *et al.*, 2016 ▸; McKee *et al.*, 2018 ▸). *C. difficile* has two TFP gene clusters, with the primary cluster encoding all of the proteins required for functional pili: the major pilin PilA1; the pre-pilin peptidase PilD; the membrane-associated proteins PilC, PilMN and PilO; the assembly and disassembly ATPases PilB and PilT; and PilU, PilV and PilK, which are known as minor pilins. PilA1 is thought to assemble into long pili filaments, which are decorated by the minor pilins. Pilin proteins can be identified by a highly conserved signal-peptide sequence (Imam *et al.*, 2011 ▸) that is cleaved by the pre-pilin peptidase PilD before insertion into the pilin filament (Melville & Craig, 2013 ▸).

**Figure 2:**
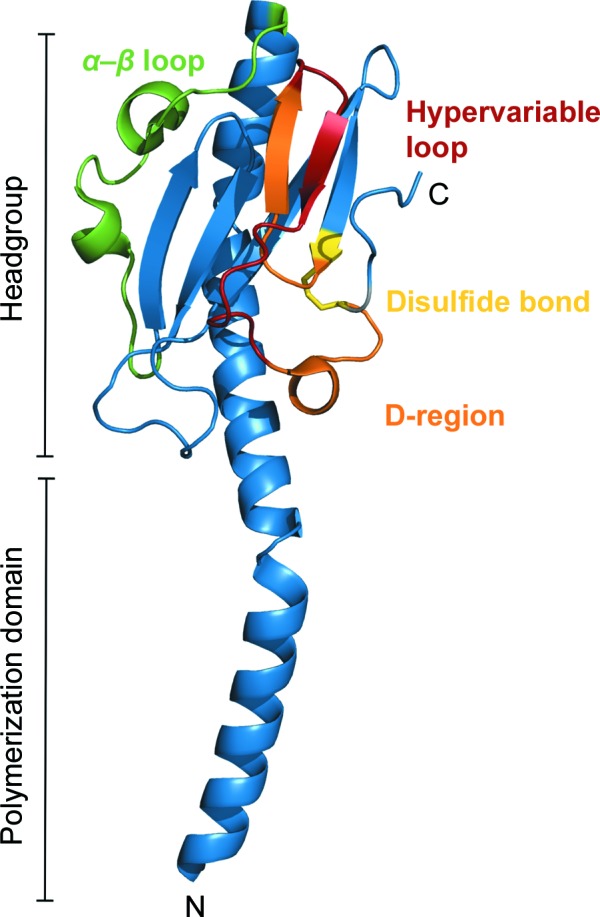
Structural features of TFP pilins. The crystal structure of full-length PilE1 from *N. gonorrhoeae* (PDB entry 2hi2; Craig *et al.*, 2006 ▸) showcasing the distinct domains of TFP pilin proteins: an N-terminal hydrophobic helix (α1) responsible for pilin polymerization, an α–β loop and a D-region. In PilE1 and other TFP pilin structures from Gram-negative bacteria determined to date, the D-region is delimited by a disulfide bond that links the termini of the D-region (Cys121–Cys151 in this case; yellow). This domain exhibits the lowest level of sequence conservation across TFP pilins and contains a hypervariable loop with the highest level of variability.

There are around 20 protein crystal structures of pilin proteins in the PDB, mostly from *Pseudomonas aeruginosa* (PDB entry 1qve; Audette *et al.*, 2004 ▸), *Neisseria gonorrhoeae* (PDB entry 2hi2; Craig *et al.*, 2006 ▸) and *N. meningitis* (PDB entry 2opd; Helaine *et al.*, 2007 ▸). Pilins exhibit a long N-terminal α-helix (α1) that forms a polymerization stalk domain, and a globular C-terminal domain, described as the headgroup, that consists of an α–β region linking the α1 helix to a β-sheet of at least three strands (Fig. 2 ▸; Craig & Li, 2008 ▸). A highly variable region, known as the D-region, is located at the C-terminus of the headgroup; it is predicted to be the most solvent-accessible element and can form interactions with host-cell surfaces (Craig & Li, 2008 ▸). The D-region is also delimited by a disulfide bridge in the TFP pilin structures from Gram-negative bacteria determined to date (Craig & Li, 2008 ▸).

Our attempts to determine the structure of a PilA1 construct lacking part of the N-terminal α1 helix to prevent self-polymerization are used here as an example of molecular-replacement approaches.

## 2. Methods   

### 2.1. Recombinant protein production and purification   

A PilA1 construct from *C. difficile* strain R20291 lacking the first 34 amino acids (PilA1Δ1–34, corresponding to 139 of 173 residues) was donated by Edward Couchman (N. Fairweather group, Imperial College London, England). CD3355 from R20291 genomic DNA was amplified and inserted into an empty pET-28a vector such that it was expressed with a non­cleavable N-terminal 6×His tag. PilA1Δ1–34 was expressed in *Escherichia coli* Rosetta cells by inoculating 1 l Luria–Bertani (LB) medium supplemented with 50 mg ml^−1^ kanamycin and 30 mg ml^−1^ chloramphenicol with 10 ml of an overnight culture (100 ml LB with the same antibiotics). The cells were grown at 37°C until an optical density at 600 nm of 0.4–0.6 was reached, at which point cultures were induced with isopropyl β-d-1-thiogalactopyranoside at a final concentration of 1 m*M*. Protein production was carried out at 20°C with agitation at 180 rev min^−1^ in an incubator for ∼20 h. The cells were harvested by centrifugation at 3036*g* for 30 min, resuspended in 20 m*M* Tris–HCl pH 8.0, 500 m*M* NaCl, centrifuged at 3011*g* for a further 10 min and the supernatant was discarded. The cell extracts were purified by nickel-affinity purification on a 5 ml HisTrap column (GE Healthcare) pre-equilibrated with 50 m*M* Tris–HCl pH 8.0, 500 m*M* NaCl. His-tagged protein was collected in 2 ml fractions during elution with 50, 125, 250, 375 and 500 m*M* imidazole in five steps of 15 ml. Peak fractions were analyzed by SDS–PAGE, pooled and the volume was reduced to ∼2 ml using a 3 kDa molecular-weight cutoff concentrator (Amicon). The sample was loaded onto a Superdex 75 16/600 size-exclusion column (GE Healthcare) pre-equilibrated with 20 m*M* Tris–HCl pH 8.0, 150 m*M* NaCl and connected to an ÄKTA pure FPLC system (GE Healthcare). Peak-containing fractions were analyzed by SDS–PAGE and those containing pure protein were pooled and concentrated to 16 mg ml^−1^ for use in crystallization trials.

### 2.2. Crystallization   

Sitting drops of PilA1Δ1–34 were dispensed in 1:1 and 2:1 protein:crystallization solution ratios in MRC crystallization plates with a Mosquito dispensing robot (TTP Labtech) using several commercial crystallization screens. Crystals measuring 150 × 150 × 100 µm were obtained after four days of incubation at 20°C in 1.6 *M* sodium citrate pH 6.5, a condition that provides cryoprotection (Bujacz *et al.*, 2010 ▸). Crystals were harvested in nylon loops and directly flash-cooled in liquid nitrogen for subsequent synchrotron data collection (Garman & Owen, 2006 ▸).

### 2.3. X-ray diffraction data collection   

Data sets were collected from four independent native crystals on the fixed-wavelength (λ = 0.92 Å) beamline I04-1 at Diamond Light Source using a Dectris PILATUS 2M pixel-array detector. Test images of each crystal at 0° and 90° were recorded and *iMosflm* (Battye *et al.*, 2011 ▸) was used to index the data and determine the likely Bravais lattice and unit-cell parameters of the crystal. The strategy function of *iMosflm* was utilized to determine the optimum starting position of the crystal for full, complete native data-set collection. The test images also informed the detector distance settings by visual assessment of the maximum resolution of spots on the test images. The detector distance was set so that the resolution at the edge of the detector was 1.5 Å. During the diffraction experiment, the crystal was rotated 200° with an exposure time of 0.5 s and each image was recorded over an oscillation of 0.1°. Such a strategy enabled the collection of a native data set with high multiplicity and completeness in order to simplify the structure-solution pipeline (Table 1 ▸).

### 2.4. X-ray diffraction data processing   

Data were indexed and integrated using *DIALS* (Gildea *et al.*, 2014 ▸), which automatically selected space group *P*4_1_2_1_2. It should be noted that this is related to space group *P*4_3_2_1_2, its enantiomer, by an inversion at the origin (Shmueli, 2010 ▸) and this ambiguity cannot be resolved at this stage. The data were then imported into a *CCP*4 project using the *CCP*4*i*2 GUI (Potterton *et al.*, 2018 ▸) before scaling using *AIMLESS* (Evans *et al.*, 2011 ▸) (Table 1 ▸). The resolution cutoff was determined by examining the CC_1/2_ and the *I*/σ(*I*) in the outer shell to a resolution where the CC_1/2_ was 0.4 (Karplus & Diederichs, 2015 ▸), combined with an *I*/σ(*I*) of over 0.5 (Evans & Murshudov, 2013 ▸). Assessment of the probabilities of the space-group selection based on systematic absences as calculated by *POINTLESS* (Evans, 2006 ▸), performed as part of the *AIMLESS* pipeline, did not solve the space-group ambiguity. For both *P*4_3_2_1_2 and *P*4_1_2_1_2 the probability based on systematic absences was 0.864, and therefore only solving the phase problem and assessing the resulting electron-density maps will enable the identification of the correct space group. Systematic absences allow discrimination between possibilities within a Laue group, but do not allow the distinction of different enantiomers.

### 2.5. Structure determination, model building and validation   

Molecular replacement was carried out within *CCP*4*i*2 (Potterton *et al.*, 2018 ▸) using *Phaser* (McCoy *et al.*, 2018 ▸) after manual model preparation using *Sculptor* (Bunkóczi & Read, 2011 ▸) and *Coot* (Emsley *et al.*, 2010 ▸). *Ab initio* MR with *ARCIMBOLDO_LITE* (Millán *et al.*, 2015 ▸) provided a correct solution, from which a model was built using *Buccaneer* (Cowtan, 2006 ▸). After iterative cycles of model building and refinement with *Coot* (Emsley *et al.*, 2010 ▸) and *REFMAC* (Murshudov *et al.*, 2011 ▸), the model was validated using *MolProbity* (Williams *et al.*, 2018 ▸). Details of these processes are described below and refinement statistics are given in Table 2 ▸.

## 3. A manual approach   

Here, we outline a simple manual approach to molecular replacement (Fig. 1 ▸) as initially attempted to determine the structure of PilA1.

### 3.1. Search for homologues   

Identifying structural models of sequence-related proteins for use as search models is the first step in the molecular-replacement process. Published structures are stored in the Protein Data Bank (PDB), and here we utilize the European instance of the database, PDBe (https://www.ebi.ac.uk/pdbe/; Armstrong *et al.*, 2020 ▸). PDBe provides two methods to perform a search of the PDB: by peptide sequence, a simple *FASTA* sequence search and the more sophisticated *PHMMER* approach (http://hmmer.org). Other tools such as *HHpred* (Zimmermann *et al.*, 2018 ▸), available as part of the MPI Bioinformatics Toolkit (https://toolkit.tuebingen.mpg.de/), can also be particularly powerful for identifying subunits within a target sequence for which there are known structures.

In these searches, the full-length sequence of PilA1 was used as the search query to ensure the greatest coverage. Using the *PHMMER* approach at PDBe, a number of structural models were identified with a sequence identity of up to 43% that could be used as search models. The full-length sequence of PilA1 was also submitted to the *HHpred* server and a number of fragments for which there are known structures were identified which covered the PilA1 sequence to varying degrees. Additionally, consideration should be made of structures identified during a literature search. While their sequence identity might be low, two proteins may display a greater structural identity and such examples could help to assemble a useful search model. In the case of PilA1, this is particularly stark as pilins, which are also highly related to pseudopilins (Melville & Craig, 2013 ▸), contain common structural motifs including a lengthy N-terminal α-helix and a globular region (Fig. 2 ▸). Using this knowledge of pilins and their common structural features allowed us to identify other potential search models within known pilin and pseudopilin structures, despite low sequence identity. This is a good example of a situation in which applying prior information on the biological properties of the protein of interest increases the pool of structures available to build a good-quality search model that may not show sequence identity above standard thresholds.

The results from both the *PHMMER* and *HHpred* searches were collated and assessed for their suitability. A summary of the models identified and their corresponding sequence identity to PilA1 are summarized in Table 3 ▸. A subset of these results with higher similarity were chosen as initial search models; these included PDB entries 4ixj and 2opd. PDB entry 4ixj represents a minor pilin from *C. difficile* (Piepenbrink *et al.*, 2014 ▸), PilJ, which has 20% identity to the PilA1 sequence. PDB entry 2opd is a pilin from *N. meningitidis* (Helaine *et al.*, 2007 ▸) that displays 38% sequence identity to the PilA1 sequence.

### 3.2. Model preparation   

A selection of tools for molecular-replacement model preparation are available in *CCP*4*i*2. These include *CCP*4*mg* and *Coot* (McNicholas *et al.*, 2011 ▸; Emsley *et al.*, 2010 ▸) for selecting the components of a search model and directly outputting them to a molecular-replacement process, while *CHAINSAW* is used for mutating and truncating a search model using sequence alignment with the target sequence (Stein, 2008 ▸). In this case, *Sculptor* was the chosen tool and is outlined in more detail in Bunkóczi & Read (2011 ▸).

The models identified during the search for homologues were downloaded from PDBe in PDB format. They were opened in *Coot* and chain *A* of each model was selected and saved in PDB format; this ensured that the file only contained chain *A* and no additional molecules such as waters or ligands. Within *CCP*4*i*2, a similar strategy of selecting only protein atoms from a specific chain is also available. The file produced in *Coot* was then imported into *Sculptor* in *CCP*4*i*2, a tool that can aid the success of molecular replacement by editing the model based on sequence similarity, mutating and/or removing unreliable regions (Bunkóczi & Read, 2011 ▸). To run *Sculptor* on a model structure, both the search model (search PDB) and a peptide-sequence alignment of the target and search proteins must be provided. A suitable alignment can be performed using *ClustalW* (Larkin *et al.*, 2007 ▸), which is available in the *CCP*4*i*2 suite as part of the model-preparation tab of the tasks menu. The target and related sequences are inputted in FASTA format and an alignment file (in ALN format) is generated, which is then available within the *CCP*4*i*2 *Sculptor* input form. The search model output by *Sculptor* is now ready for use in a molecular-replacement process.

To create ensembles containing multiple structures, search models edited to contain a single chain were loaded into *Coot* and aligned using the secondary-structure matching tool (*SSM*). The coordinates of the aligned structures were then saved in PDB format. These edited search models were then input into the ensemble builder for *Phaser* in *CCP*4*i*2. The peptide sequences of these models were aligned as described previously and were also added to the input form.

### 3.3. Molecular replacement   

Here, we will focus on *Phaser* (McCoy *et al.*, 2007 ▸) as the main tool for MR within *CCP*4*i*2. The input parameters for basic MR using *Phaser* require a reflection file, information about the composition of the asymmetric unit and a search model. In *CCP*4*i*2, the reflection file is selected from the output from *AIMLESS*. The composition of the asymmetric unit is determined by inputting the sequence of the target protein; a job is then run to calculate the Matthews coefficient, which allows an estimation of the likely number of molecules in the asymmetric unit. In the case of PilA1, the Matthews coefficient was ambiguous between either three or four possible copies in the asymmetric unit. Finally, the search model, provided as a PDB file containing either a single search model or an ensemble of search models, is selected. In this section, *Phaser* can also be given information regarding the similarity of the search models to the target protein either as a sequence-identity score or a root-mean-squared (r.m.s.) value, indicating the expected deviation between the target and search structures (Supplementary Fig. S1).

At the end of the process, *Phaser* provides two key scores that must be assessed to determine whether a correct solution with phases that allow the calculation of an interpretable electron-density map has been obtained: the TFZ score and the LLG. A TFZ (translation-function *Z*-score) value above 8 indicates that the solution has definitely been found, while a score of 7 or lower suggests that it is increasingly less likely that a correct solution has been obtained. During the *Phaser* process, the LLG is also a good indicator of whether the solution is correct, and this value should increase greatly as a correct solution is found (usually to greater than 100). Both of these values are displayed in the *Phaser* job window in *CCP*4*i*2 as the job runs and can be found in the results window once the process has completed (Supplementary Fig. S2). In the case of PilA1 using our ensemble, these values are a TFZ of 5.7 and a final LLG of 29, indicating that this was not a correct solution. When viewed in *Coot* the map was discontinuous, also confirming that the phase problem had not been solved. Owing to the space-group ambiguity, the search was carried out in *Phaser* selecting all possible enantiomorphs. The selected solution was in space group *P*4_3_2_1_2, the enantiomorph of *P*4_1_2_1_2, but the TFZ scores for other possibilities were similarly low. In any case, this is an ambiguity that would only be clearly resolved if the phase problem had been correctly solved.

Unfortunately, in the case of PilA1, we have not successfully found a solution using this method of MR. However, this is a useful approach to use, especially if more similar structural homologues are available. Fortunately, other methods are available which utilize MR without the need to collect any further diffraction data, such as experimental phasing data.

### 3.4. *Ab initio* MR   

Molecular replacement without search models is becoming ever more possible on a desktop computer, using programs such as *AMPLE* and *ARCIMBOLDO*. *ARCIMBOLDO* uses a fragment-based approach, placing short fragments from secondary-structure predictions to determine the initial phases (Millán *et al.*, 2015 ▸). *ARCIMBOLDO* uses *Phaser* to place these fragments before density modification using *SHELXE* (Sheldrick, 2010 ▸). PilA1 is a promising candidate for *ARCIMBOLDO*, with the full-length PilA1 predicted to contain an extended N-terminal α-helix of approximately 60 residues and the diffraction data collected to a resolution of greater than 2 Å.

Tools such as *PSIPRED* (http://bioinf.cs.ucl.ac.uk/psipred/) and *JPred* (http://www.compbio.dundee.ac.uk/jpred/) allow secondary-structure predictions when provided with a peptide sequence (Buchan & Jones, 2019 ▸; Jones, 1999 ▸; Drozdetskiy *et al.*, 2015 ▸). The output of a *PSIPRED* analysis of the truncated construct of PilA1 used here predicts a 30-residue-long α-helix, as expected based on our knowledge of TFP pilins. The *ARCIMBOLDO_LITE* distribution can now be used on a single multi-core machine and is distributed with *CCP*4*i*2. The information required to run a job includes scaled reflection data (MTZ format), the molecular mass of the target protein and the number of copies in the asymmetric unit. As the PilA1 crystals are calculated to contain either three or four copies in the asymmetric unit, two separate processes were run searching for three or four copies of an α-helix containing 30 residues.

**Figure 3:**
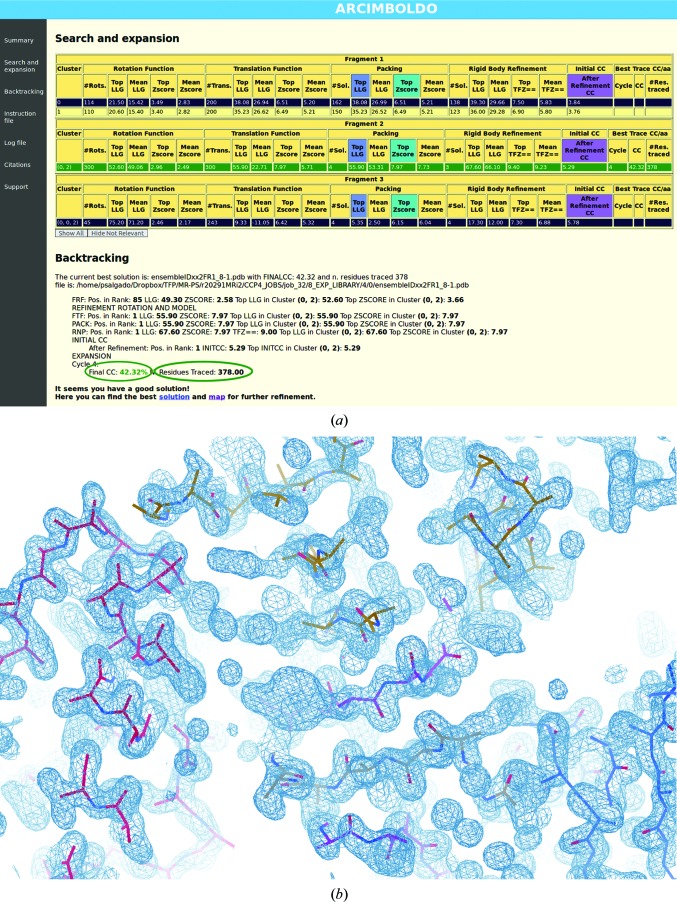
*Ab initio* MR with *ARCIMBOLDO_LITE*. (*a*) HTML-based *ARCIMBOLDO_LITE* output, showing that three 30-residue α-helical fragments have been found and the corresponding statistics. The overall correlation coefficient (CC) was 43.2%, as highlighted, and 378 residues have been traced. (*b*) Electron-density map (blue chicken wire) at a 1.5σ contour level showing that well defined density and clearly identifiable side chains were obtained. The traced residues are built as polyalanine chains (stick representation) in *ARCIMBOLDO_LITE*, which fitted well into the electron-density map.

In the case of PilA1, after approximately 20 h of run time on a modest desktop machine, *ARCIMBOLDO_LITE* was able to provide a solution. An electron-density map, a PDB file of the backbone of the best solution and an HTML-based report were output (Fig. 3 ▸ *a*). In line with the *SHELX* package, the success of the process is assessed using a correlation coefficient (CC). A correct solution is likely to have been found when the CC is greater than 25% (Usón & Sheldrick, 2018 ▸). The final CC for the PilA1 process is 42.3%, indicating that a correct solution has been determined (Fig. 1 ▸ *a*). Additionally, 378 out of 432 residues were built, including the key structural features of TFP (details in Fig. 2 ▸): one N-terminal α-helix from each molecule (each of approximately 30 residues), as well as the β-sheet characteristic of the head domain. On inspection of the electron-density map, good continuity is observed, a further indication that the phases had been correctly calculated and a solution had been found (Fig. 3 ▸ *b*).

In the case of PilA1, *ab initio* MR methods were successful in solving the phase problem, rather than using a search model that was based on sequence homology. As such, the output of *ARCIMBOLDO* was used for model building and refinement.

### 3.5. Model building, refinement and validation   

Two model-building tools are provided in *CCP*4*i*2: *ARP*/*wARP* (Chojnowski *et al.*, 2019 ▸) and *Buccaneer* (Cowtan, 2006 ▸). For the purposes of this workflow, we used *Buccaneer* to build upon the polyalanine model that was generated by *ARCIMBOLDO*. *Buccaneer* has two input modes in *CCP*4*i*2 that are tailored to phase problems that have been solved using experimental phasing or using molecular replacement. For PilA1, we selected the molecular-replacement mode and therefore we must input the search model that was used for structure determination. In this case, we can input the polyalanine model generated by *ARCIMBOLDO_LITE*. We must also input the scaled reflections and the free *R* set that were generated by *AIMLESS*. Finally, we must define the crystal contents by inputting the target sequence. These are the only essential pieces of information that *Buccaneer* requires to run the process, but a large number of more advanced parameters can also be input. For the case of PilA1, the default values were used. After model building, *Buccaneer* also utilizes *REFMAC* (Murshudov *et al.*, 2011 ▸) to perform an initial round of refinement. As well as visually inspecting the resulting output, it is also important to note the statistics of the completed job, including the number of residues that have been built as well as the refinement measures *R*_work_ and *R*_free_. These initial refinement statistics may be high (∼0.5) but will decrease with further rounds of refinement, which involves iterative cycles of automated refinement using *REFMAC* (Murshudov *et al.*, 2011 ▸), followed by inspection and manual adjustment in *Coot*. As the resolution of the PilA1Δ1–34 data (1.65 Å) is borderline for the use of anisotropic *B* factors in refinement (Merritt, 2012 ▸), an isotropic refinement protocol was used throughout. *Coot* was used to refine local bond angles, check the sequence register with the electron density, add alternate side-chain conformations and add water molecules. After several rounds of refinement, *R*_work_ and *R*_free_ values of 19.4% and 21.4%, respectively, were achieved (Brünger, 1992 ▸, 1993 ▸).

Structure validation was performed using *MolProbity* (Williams *et al.*, 2018 ▸). This software also identified and flipped any aspartate, glutamate or histidine residues that clashed and added riding hydrogens so that hydrogen-bonding potential can be assessed (Chen *et al.*, 2010 ▸). Clashing side chains, unfavoured rotamers and Ramachandran outliers were identified by *MolProbity* and problematic residues were inspected and manually fixed using *Coot* whenever the electron-density maps failed to support the unusual conformations. After a further round of *REFMAC* refinement and assessment in *MolProbity*, it was noted that there were still a number of bond lengths and angles with root-mean-squared deviations (r.m.s.d.s) outside the acceptable ranges (Rossmann & Arnold, 2001 ▸). A weighting term was applied in *REFMAC*, also under restraints in the input form, starting at 0.1 and incremented in steps of 0.5. The output of these was assessed in *MolProbity* until there were no outliers unless justified by the electron-density maps and the *R*_work_ and *R*_free_ values were closely observed to ensure that there was no bias occurring from the weighting. A final weighting term of 0.1 was chosen.

Final CIF files were prepared using the *CCP*4*i*2 interface under the deposition section of the task menu for analysis using the PDBe validation server (https://validate-pdbe.wwpdb.org/). Once validated, the files were uploaded to the PDBe deposition server (https://deposit-pdbe.wwpdb.org/) with PDB identifier 6t8s.

## 4. PilA1 structure   

**Figure 4:**
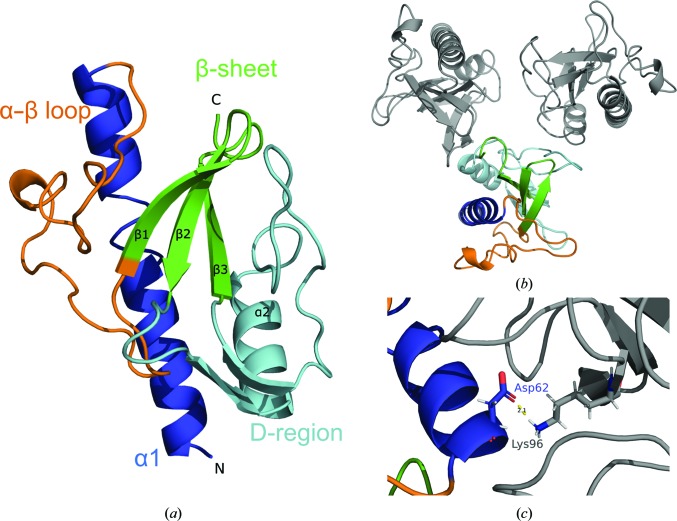
R20291 PilA1 structure. (*a*) Chain *A* of R20291 PilA1Δ1–34 (cartoon) contains the structural regions associated with TFP pilin structures: a long α1 helix (dark blue) followed by an α–β loop that connects the long α-helix and an antiparallel β-sheet (green). The variable D-region (cyan) contains a shorter α-helix of ten residues in length, but is mostly composed of flexible loops. (*b*) Top view of the three molecules observed in the asymmetric unit of R20291 PilA1Δ1–34, showing potential pilin–pilin interfaces. (*c*) Analysis using *PISA* indicated a possible interface between R20291 PilA1Δ1–34 molecules via an Asp62–Lys96 salt bridge.

The structure of R20291 PilA1Δ1–34 (Fig. 4 ▸) is typical of the TFP pilins, containing a long N-terminal α-helix (α1) which is linked by an α–β loop to an antiparallel β-sheet (β1–β2–β3) that encapsulates the D-region containing a shorter α-helix (α2), with the rest of this subdomain largely formed of loop regions. As there are no cysteine residues in PilA1, it lacks the disulfide bridge that delimits the D-region of Gram-negative pilins.

The crystal structures of PilA1 from R20291 and two other strains were published in 2015, and although these structures included a maltose-binding protein (MBP) tag, the structure of the R20291 PilA1 monomer was essentially the same (Piepenbrink *et al.*, 2015 ▸). The core r.m.s.d. between our model and the published structure, as calculated using the secondary-structure matching tool in *Coot*, was 0.89 Å. However, Piepenbrink and coworkers determined the structures of MBP-PilA1 fusions, while the structure described here contains a much shorter 6×His tag, allowing us to observe PilA1 multimers which provide possible PilA1 interfaces. There are three molecules in the asymmetric unit and interactions within this arrangement could be biologically relevant for pilus formation, so the coordinates were submitted to the *Protein Interfaces, Surfaces and Assemblies* (*PISA*) server (Krissinel & Henrick, 2007 ▸) for investigation. *PISA* scores interfaces between molecules using a complex-formation significance score (CSS), and is used to assess whether molecular interfaces are biologically significant or a product of crystallization: 0.0 indicates no biological significance and a value of 1.0 indicates significant complex formation (Krissinel & Henrick, 2007 ▸). A summary of the interfaces detected by *PISA* is presented in Supplementary Table S1. Of particular interest was a salt bridge between Asp62 and Lys96 in neighbouring R20291 PilA1Δ1–34 molecules (Fig. 4 ▸), part of an interface with a calculated surface area representing 7% of the solvent-accessible area of the pilin. A number of hydrogen-bonding interactions were also predicted to contribute to this interface, indicating that it could be biologically relevant. Moreover, attempts to mutate either residue to alanine resulted in insoluble or no recombinant protein being produced, suggesting that these residues are important for protein stability. Interestingly, this interaction had not been observed in the published structural models (Piepenbrink *et al.*, 2014 ▸, 2015 ▸) as the presence of the large MBP tag prevented any direct PilA1–PilA1 interactions. Instead, the authors of these studies suggested that a different interaction would be relevant for TFP filament formation based on the models of filament formation proposed for the TFP filaments from *N. meningitidis*, *N. gonorrhoeae* and *Vibrio cholerae* (Hartung *et al.*, 2011 ▸; Craig & Li, 2008 ▸; Craig *et al.*, 2003 ▸, 2006 ▸). These models were built using crystallographic and NMR structures of the pilin proteins and electron-microscopy data of the full filament structure to fit the pilin units (Craig & Li, 2008 ▸). Such models place the extended, hydrophobic N-terminal α-helix within the core of the filament, interacting with other pilin units via hydrophobic interactions. Based on the similarities between PilA1 and these structures, Piepenbrink *et al.* (2015 ▸) proposed a model for TFP filament formation in *C. difficile* in which a salt bridge between PilA1 units via Lys30 and Glu75 would be equivalent to that observed in the TcpA filament model. However, this salt bridge is not observed in our direct PilA1–PilA1 interactions.

As part of our analysis of the PilA1 structure, we submitted our model to the *PDBeFold* (Krissinel & Henrick, 2004 ▸) and *DALI* (Holm & Rosenström, 2010 ▸) servers to identify similar structures in the PDB. This search readily retrieved structures of several TFP pilins, such as the full-length PilE1 (PDB entry 2hi2) from *N. gonorrhoeae* (Craig *et al.*, 2006 ▸) that shared 39% sequence identity with the *C. difficile* protein. It also retrieved the structures of the major pilins from other Gram-positive bacteria such as TcpA from *V. cholerae* (PDB entries 1oqv and 3hrv; Craig *et al.*, 2003 ▸; Lim *et al.*, 2010 ▸), CofA from *E. coli* (PDB entry 3s0t; Kolappan *et al.*, 2012 ▸) and PilS from *Salmonella typhi* (PDB entry 3fhu; Balakrishna *et al.*, 2009 ▸). Interestingly, the structures of type II secretion system (TIISS) pseudopilins from Gram-negative bacteria were also retrieved, such as PulG (PDB entry 1t92) from *Klebsiella pneumoniae* (Köhler *et al.*, 2004 ▸), EspG from *V. cholerae* (PDB entry 4lw9; F. S. Vago, K. Raghunathan, J. C. Jens, W. J. Wedemeyer, M. Bagdasarian, J. S. Brunzelle & D. N. Arvidson, unpublished work) and GspG from *E. coli* (PDB entry 3g20; Korotkov *et al.*, 2009 ▸). Pseudopilins are part of the TIISS, are similar in architecture to TFP and export pilin-like units; however, they do not readily form filaments as observed in TFP (Korotkov *et al.*, 2012 ▸). All structures share the commonly observed fold of TFP, including the N-terminal α-helix (α1) connected to the head domain and the variable D-region at the C-termini (Fig. 2 ▸). Whether Gram-positive TFP pilins are functionally more similar to the TIISS pseudopilins or the Gram-negative pilins remains to be determined once full-length pilin and filament structures have been determined. It will therefore be important to determine the structures of the full-length PilA1 and other minor pilins, as well as investigate the interactions between these proteins, to elucidate pili filament formation. Together, this information would allow us to start unravelling the mechanisms underlying TFP formation and assembly in *C. difficile*.

## 5. Conclusion   

Despite collecting high-resolution data for R20291 PilA1Δ1–34, it was not possible to calculate phases and an interpretable electron-density map using our manually edited search models based on known pilin structures. As the native data set for R20291 PilA1Δ1–34 had high resolution (1.65 Å), high completeness, low *R*_meas_ and high *I*/σ(*I*), it was an ideal candidate for *ab initio* phasing using *ARCIMBOLDO* (Millán *et al.*, 2015 ▸). As our example shows, using a careful approach to molecular-replacement pipelines and exploring available options, combined with prior knowledge of both the biology and structural characteristics of the protein of interest, can lead to solution of the phase problem and the determination of novel protein structures.

## Supplementary Material

Supplementary Table and Figures. DOI: 10.1107/S2059798320000467/ip5006sup1.pdf

PDB reference: PilA1, 6t8s

## Figures and Tables

**Table 1:** Data-processing statistics for PilA1 Values in parentheses are for the outer shell.

X-ray source	Beamline I04-1, Diamond Light Source
Wavelength (Å)	0.92
Resolution range (Å)	51.21–1.65
Unit-cell parameters	
*a* = *b*, *c* (Å)	102.57, 104.34
α = β = γ (°)	90
Space group	*P*4_1_2_1_2 or *P*4_3_2_1_2
Total No. of reflections	964312 (39779)
No. of unique reflections	67404 (3396)
〈*I*/σ(*I*)〉	12.0 (0.6)
CC_1/2_	0.999 (0.390)
*R*_meas_	0.142 (3.961) [0.05†]
Multiplicity	14.3 (11.7)
Completeness (%)	100.0 (100.0)
Mosaicity (°)	0.12
Wilson *B* factor (Å^2^)	27.1

† Low-resolution shell.

**Table 2:** Refinement statistics for PilA1 Ramachandran statistics and *MolProbity* clashscore were obtained using *MolProbity* (Williams *et al.*, 2018 ▸).

Space group	*P*4_1_2_1_2
*R*_work_ (%)	19.4
*R*_free_ (%)	21.4
No. of non-H atoms	
Protein	3069
Solvent	254
No. of residues	400
R.m.s. deviations	
Bond angles (°)	1.30
Bond lengths (Å)	0.01
Average *B* factor (Å^2^)	
Overall	28.00
Protein	28.53
Water	34.38
Ramachandran plot	
Most favoured regions (%)	97.21
Allowed regions (%)	2.57
Outliers (%)	0
Clashscore	0.48
*MolProbity* clashscore	0.81

**Table 3:** Related homologues used to build the search ensemble and methods of identification

Protein, organism	PDB code	Sequence identity (%)	Method of identification
PilX, *Neisseria meningitidis*	2opd	38.46	PDBe *BLAST*, *HHPred*
Pseudopilin, *Escherichia coli*	3g20	28.30	PDBe *BLAST*, *HHPred*
PulG, *Klebsiella pneumoniae*	1t92	27.18	PDBe *BLAST*, *HHPred*
PilJ, *Clostridioides difficile*	4ixj	19.86	PDBe *BLAST*, *HHPred*
PilE, *Francisella tularensis*	3soj	14.42	Literature review
PilA, *Acinetobacter baumannii*	5vaw	13.49	Literature review
FimU, *Pseudomonas aeruginosa*	4ipu	13.51	*HHPred*

## References

[bb1] Armstrong, D. R., Berrisford, J. M., Conroy, M. J., Gutmanas, A., Anyango, S., Choudhary, P., Clark, A. R., Dana, J. M., Deshpande, M., Dunlop, R., Gane, P., Gáborová, R., Gupta, D., Haslam, P., Koča, J., Mak, L., Mir, S., Mukhopadhyay, A., Nadzirin, N., Nair, S., Paysan-Lafosse, T., Pravda, L., Sehnal, D., Salih, O., Smart, O., Tolchard, J., Varadi, M., Svobodova-Vařeková, R., Zaki, H., Kleywegt, G. J. & Velankar, S. (2020). *Nucleic Acids Res.* **48**, D335–D343. 10.1093/nar/gkz990 PMC7145656 31691821

[bb80] Audette, G. F., Irvin, R. T. & Hazes, B. (2004). *Biochemistry*, **43**, 11427–11435. 10.1021/bi048957s 15350129

[bb2] Aurelius, O., Duman, R., El Omari, K., Mykhaylyk, V. & Wagner, A. (2017). *Nucl. Instrum. Methods Phys. Res. B*, **411**, 12–16. 10.1016/j.nimb.2016.12.005 PMC5727679 29276323

[bb90] Balakrishna, A. M., Saxena, A. M., Mok, H. Y. & Swaminathan, K. (2009). *Proteins*, **77**, 253–261. 10.1002/prot.22500 19626704

[bb3] Basu, S., Olieric, V., Leonarski, F., Matsugaki, N., Kawano, Y., Takashi, T., Huang, C.-Y., Yamada, Y., Vera, L., Olieric, N., Basquin, J., Wojdyla, J. A., Bunk, O., Diederichs, K., Yamamoto, M. & Wang, M. (2019). *IUCrJ*, **6**, 373–386. 10.1107/S2052252519002756 PMC6503925 31098019

[bb4] Battye, T. G. G., Kontogiannis, L., Johnson, O., Powell, H. R. & Leslie, A. G. W. (2011). *Acta Cryst.* D**67**, 271–281. 10.1107/S0907444910048675 PMC3069742 21460445

[bb5] Bent, A. F., Mann, G., Houssen, W. E., Mykhaylyk, V., Duman, R., Thomas, L., Jaspars, M., Wagner, A. & Naismith, J. H. (2016). *Acta Cryst.* D**72**, 1174–1180. 10.1107/S2059798316015850 PMC5108345 27841750

[bb6] Bibby, J., Keegan, R. M., Mayans, O., Winn, M. D. & Rigden, D. J. (2012). *Acta Cryst.* D**68**, 1622–1631. 10.1107/S0907444912039194 23151627

[bb7] Brünger, A. T. (1992). *Nature*, **355**, 472–475. 10.1038/355472a0 18481394

[bb8] Brünger, A. T. (1993). *Acta Cryst.* D**49**, 24–36. 10.1107/S0907444992007352 15299543

[bb9] Buchan, D. W. A. & Jones, D. T. (2019). *Nucleic Acids Res.* **47**, W402–W407. 10.1093/nar/gkz297 PMC6602445 31251384

[bb10] Bujacz, G., Wrzesniewska, B. & Bujacz, A. (2010). *Acta Cryst.* D**66**, 789–796. 10.1107/S0907444910015416 20606259

[bb11] Bunkóczi, G. & Read, R. J. (2011). *Acta Cryst.* D**67**, 303–312. 10.1107/S0907444910051218 PMC3069745 21460448

[bb12] Chen, V. B., Arendall, W. B., Headd, J. J., Keedy, D. A., Immormino, R. M., Kapral, G. J., Murray, L. W., Richardson, J. S. & Richardson, D. C. (2010). *Acta Cryst.* D**66**, 12–21. 10.1107/S0907444909042073 PMC2803126 20057044

[bb13] Chojnowski, G., Pereira, J. & Lamzin, V. S. (2019). *Acta Cryst.* D**75**, 753–763. 10.1107/S2059798319009392 PMC6677015 31373574

[bb14] Cowtan, K. (2006). *Acta Cryst.* D**62**, 1002–1011. 10.1107/S0907444906022116 16929101

[bb15] Craig, L. & Li, J. (2008). *Curr. Opin. Struct. Biol.* **18**, 267–277. 10.1016/j.sbi.2007.12.009 PMC2442734 18249533

[bb16] Craig, L., Taylor, R. K., Pique, M. E., Adair, B. D., Arvai, A. S., Singh, M., Lloyd, S. J., Shin, D. S., Getzoff, E. D., Yeager, M., Forest, K. T. & Tainer, J. A. (2003). *Mol. Cell*, **11**, 1139–1150. 10.1016/s1097-2765(03)00170-9 12769840

[bb17] Craig, L., Volkmann, N., Arvai, A. S., Pique, M. E., Yeager, M., Egelman, E. H. & Tainer, J. A. (2006). *Mol. Cell*, **23**, 651–662. 10.1016/j.molcel.2006.07.004 16949362

[bb18] Drozdetskiy, A., Cole, C., Procter, J. & Barton, G. J. (2015). *Nucleic Acids Res.* **43**, W389–W394. 10.1093/nar/gkv332 PMC4489285 25883141

[bb19] Emsley, P., Lohkamp, B., Scott, W. G. & Cowtan, K. (2010). *Acta Cryst.* D**66**, 486–501. 10.1107/S0907444910007493 PMC2852313 20383002

[bb20] Evans, G., Axford, D. & Owen, R. L. (2011). *Acta Cryst.* D**67**, 261–270. 10.1107/S0907444911007608 PMC3069741 21460444

[bb21] Evans, P. (2006). *Acta Cryst.* D**62**, 72–82. 10.1107/S0907444905036693 16369096

[bb22] Evans, P. & McCoy, A. (2008). *Acta Cryst.* D**64**, 1–10. 10.1107/S0907444907051554 PMC2394790 18094461

[bb23] Evans, P. R. & Murshudov, G. N. (2013). *Acta Cryst.* D**69**, 1204–1214. 10.1107/S0907444913000061 PMC3689523 23793146

[bb24] Fedosyuk, S., Bezerra, G. A., Radakovics, K., Smith, T. K., Sammito, M., Bobik, N., Round, A., Ten Eyck, L. F., Djinović-Carugo, K., Usón, I. & Skern, T. (2016). *PLoS Pathog.* **12**, e1006079. 10.1371/journal.ppat.1006079 PMC5156371 27973613

[bb25] Garman, E. & Murray, J. W. (2003). *Acta Cryst.* D**59**, 1903–1913. 10.1107/s0907444903012794 14573944

[bb26] Garman, E. F. & Owen, R. L. (2006). *Acta Cryst.* D**62**, 32–47. 10.1107/S0907444905034207 16369092

[bb27] Gildea, R. J., Waterman, D. G., Parkhurst, J. M., Axford, D., Sutton, G., Stuart, D. I., Sauter, N. K., Evans, G. & Winter, G. (2014). *Acta Cryst.* D**70**, 2652–2666. 10.1107/S1399004714017039 PMC4188007 25286849

[bb28] Hartung, S., Arvai, A. S., Wood, T., Kolappan, S., Shin, D. S., Craig, L. & Tainer, J. A. (2011). *J. Biol. Chem.* **286**, 44254–44265. 10.1074/jbc.M111.297242 PMC3243539 22027840

[bb29] Helaine, S., Dyer, D. H., Nassif, X., Pelicic, V. & Forest, K. T. (2007). *Proc. Natl Acad. Sci. USA*, **104**, 15888–15893. 10.1073/pnas.0707581104 PMC2000383 17893339

[bb30] Holm, L. & Rosenström, P. (2010). *Nucleic Acids Res.* **38**, W545–W549. 10.1093/nar/gkq366 PMC2896194 20457744

[bb31] Huang, H., Weintraub, A., Fang, H. & Nord, C. E. (2009). *Int. J. Antimicrob. Agents*, **34**, 516–522. 10.1016/j.ijantimicag.2009.09.012 19828299

[bb32] Imam, S., Chen, Z., Roos, D. S. & Pohlschröder, M. (2011). *PLoS One*, **6**, e28919. 10.1371/journal.pone.0028919 PMC3244431 22216142

[bb33] Jones, D. T. (1999). *J. Mol. Biol.* **292**, 195–202. 10.1006/jmbi.1999.3091 10493868

[bb34] Karplus, P. A. & Diederichs, K. (2015). *Curr. Opin. Struct. Biol.* **34**, 60–68. 10.1016/j.sbi.2015.07.003 PMC4684713 26209821

[bb35] Köhler, R., Schäfer, K., Müller, S., Vignon, G., Diederichs, K., Philippsen, A., Ringler, P., Pugsley, A. P., Engel, A. & Welte, W. (2004). *Mol. Microbiol.* **54**, 647–664. 10.1111/j.1365-2958.2004.04307.x 15491357

[bb89] Kolappan, S., Roos, J., Yuen, A. S., Pierce, O. M. & Craig, L. (2012). *J. Bacteriol.* **194**, 2725–2735. 10.1128/JB.00282-12 PMC3347196 22447901

[bb36] Korotkov, K. V., Sandkvist, M. & Hol, W. G. J. (2012). *Nat. Rev. Microbiol.* **10**, 336–351. 10.1038/nrmicro2762 PMC3705712 22466878

[bb86] Korotkov, K. V., Gray, M. D., Kreger, A., Turley, S., Sandkvist, M. & Hol, W. G. J. (2009). *J. Biol. Chem.* **284**, 25466–25470. 10.1074/jbc.C109.037655 PMC2757947 19640838

[bb37] Krissinel, E. & Henrick, K. (2004). *Acta Cryst.* D**60**, 2256–2268. 10.1107/S0907444904026460 15572779

[bb38] Krissinel, E. & Henrick, K. (2007). *J. Mol. Biol.* **372**, 774–797. 10.1016/j.jmb.2007.05.022 17681537

[bb39] Krojer, T., Talon, R., Pearce, N., Collins, P., Douangamath, A., Brandao-Neto, J., Dias, A., Marsden, B. & von Delft, F. (2017). *Acta Cryst.* D**73**, 267–278. 10.1107/S2059798316020234 PMC5349439 28291762

[bb40] Larkin, M. A., Blackshields, G., Brown, N. P., Chenna, R., McGettigan, P. A., McWilliam, H., Valentin, F., Wallace, I. M., Wilm, A., Lopez, R., Thompson, J. D., Gibson, T. J. & Higgins, D. G. (2007). *Bioinformatics*, **23**, 2947–2948. 10.1093/bioinformatics/btm404 17846036

[bb41] Liebschner, D., Afonine, P. V., Baker, M. L., Bunkóczi, G., Chen, V. B., Croll, T. I., Hintze, B., Hung, L.-W., Jain, S., McCoy, A. J., Moriarty, N. W., Oeffner, R. D., Poon, B. K., Prisant, M. G., Read, R. J., Richardson, J. S., Richardson, D. C., Sammito, M. D., Sobolev, O. V., Stockwell, D. H., Terwilliger, T. C., Urzhumtsev, A. G., Videau, L. L., Williams, C. J. & Adams, P. D. (2019). *Acta Cryst.* D**75**, 861–877. 10.1107/S2059798319011471 PMC6778852 31588918

[bb84] Lim, M. S., Ng, D., Zong, Z., Arvai, A. S., Taylor, R. K., Tainer, J. A. & Craig, L. (2010). *Mol. Microbiol.* **77**, 755–770. 10.1111/j.1365-2958.2010.07244.x PMC2939829 20545841

[bb42] Long, F., Vagin, A. A., Young, P. & Murshudov, G. N. (2008). *Acta Cryst.* D**64**, 125–132. 10.1107/S0907444907050172 PMC2394813 18094476

[bb43] Maldarelli, G. A., Matz, H., Gao, S., Chen, K., Hamza, T., Yfantis, H. G., Feng, H. & Donnenberg, M. S. (2016). *J. Vaccines Vaccin.* **7**, 321. 10.4172/2157-7560.1000321 PMC4927082 27375958

[bb44] Maldarelli, G. A., Piepenbrink, K. H., Scott, A. J., Freiberg, J. A., Song, Y., Achermann, Y., Ernst, R. K., Shirtliff, M. E., Sundberg, E. J., Donnenberg, M. S. & von Rosenvinge, E. C. (2016). *Pathog. Dis.* **74**, ftw061. 10.1093/femspd/ftw061 PMC5985507 27369898

[bb45] Manetti, A. G. O., Zingaretti, C., Falugi, F., Capo, S., Bombaci, M., Bagnoli, F., Gambellini, G., Bensi, G., Mora, M., Edwards, A. M., Musser, J. M., Graviss, E. A., Telford, J. L., Grandi, G. & Margarit, I. (2007). *Mol. Microbiol.* **64**, 968–983. 10.1111/j.1365-2958.2007.05704.x 17501921

[bb46] McCoy, A. J., Grosse-Kunstleve, R. W., Adams, P. D., Winn, M. D., Storoni, L. C. & Read, R. J. (2007). *J. Appl. Cryst.* **40**, 658–674. 10.1107/S0021889807021206 PMC2483472 19461840

[bb47] McCoy, A. J., Oeffner, R. D., Millán, C., Sammito, M., Usón, I. & Read, R. J. (2018). *Acta Cryst.* D**74**, 279–289. 10.1107/S2059798318001353 PMC5892877 29652255

[bb48] McKee, R. W., Aleksanyan, N., Garrett, E. M. & Tamayo, R. (2018). *Infect. Immun.* **86**, e00943-17. 10.1128/IAI.00943-17 PMC5913833 29483294

[bb49] McNicholas, S., Potterton, E., Wilson, K. S. & Noble, M. E. M. (2011). *Acta Cryst.* D**67**, 386–394. 10.1107/S0907444911007281 PMC3069754 21460457

[bb50] Melville, S. & Craig, L. (2013). *Microbiol. Mol. Biol. Rev.* **77**, 323–341. 10.1128/MMBR.00063-12 PMC3811610 24006467

[bb51] Merritt, E. A. (2012). *Acta Cryst.* D**68**, 468–477. 10.1107/S0907444911028320 PMC3322606 22505267

[bb52] Millán, C., Sammito, M. & Usón, I. (2015). *IUCrJ*, **2**, 95–105. 10.1107/S2052252514024117 PMC4285884 25610631

[bb53] Murshudov, G. N., Skubák, P., Lebedev, A. A., Pannu, N. S., Steiner, R. A., Nicholls, R. A., Winn, M. D., Long, F. & Vagin, A. A. (2011). *Acta Cryst.* D**67**, 355–367. 10.1107/S0907444911001314 PMC3069751 21460454

[bb54] Piepenbrink, K. H., Maldarelli, G. A., de la Peña, C. F. M., Mulvey, G. L., Snyder, G. A., De Masi, L., von Rosenvinge, E. C., Günther, S., Armstrong, G. D., Donnenberg, M. S. & Sundberg, E. J. (2014). *J. Biol. Chem.* **289**, 4334–4345. 10.1074/jbc.M113.534404 PMC3924296 24362261

[bb55] Piepenbrink, K. H., Maldarelli, G. A., Martinez de la Peña, C. F., Dingle, T. C., Mulvey, G. L., Lee, A., von Rosenvinge, E., Armstrong, G. D., Donnenberg, M. S. & Sundberg, E. J. (2015). *Structure*, **23**, 385–396. 10.1016/j.str.2014.11.018 PMC4318773 25599642

[bb56] Potterton, L., Agirre, J., Ballard, C., Cowtan, K., Dodson, E., Evans, P. R., Jenkins, H. T., Keegan, R., Krissinel, E., Stevenson, K., Lebedev, A., McNicholas, S. J., Nicholls, R. A., Noble, M., Pannu, N. S., Roth, C., Sheldrick, G., Skubak, P., Turkenburg, J., Uski, V., von Delft, F., Waterman, D., Wilson, K., Winn, M. & Wojdyr, M. (2018). *Acta Cryst.* D**74**, 68–84. 10.1107/S2059798317016035 PMC5947771 29533233

[bb58] Rigden, D. J., Keegan, R. M. & Winn, M. D. (2008). *Acta Cryst.* D**64**, 1288–1291. 10.1107/S0907444908033192 19018106

[bb59] Rossmann, M. G. & Arnold, E. (2001). Editors. *International Tables for Crystallography*, Vol. *F*. Dordrecht: Kluwer Academic Publishers.

[bb60] Sammito, M., Millán, C., Rodríguez, D. D., de Ilarduya, I. M., Meindl, K., De Marino, I., Petrillo, G., Buey, R. M., de Pereda, J. M., Zeth, K., Sheldrick, G. M. & Usón, I. (2013). *Nat. Methods*, **10**, 1099–1101. 10.1038/nmeth.2644 24037245

[bb61] Sheldrick, G. M. (2010). *Acta Cryst.* D**66**, 479–485. 10.1107/S0907444909038360 PMC2852312 20383001

[bb62] Shmueli, U. (2010). Editor. *International Tables for Crystallography*, Vol. *B*, 2nd online ed. Chester: IUCr.

[bb63] Silvestri, I., Lyu, H., Fata, F., Boumis, G., Miele, A. E., Ardini, M., Ippoliti, R., Bellelli, A., Jadhav, A., Lea, W. A., Simeonov, A., Cheng, Q., Arnér, E. S. J., Thatcher, G. R. J., Petukhov, P. A., Williams, D. L. & Angelucci, F. (2018). *ACS Chem. Biol.* **13**, 2190–2202. 10.1021/acschembio.8b00349 PMC6905387 29800515

[bb64] Stein, N. (2008). *J. Appl. Cryst.* **41**, 641–643.

[bb65] Taylor, G. (2003). *Acta Cryst.* D**59**, 1881–1890. 10.1107/s0907444903017815 14573942

[bb66] Taylor, G. L. (2010). *Acta Cryst.* D**66**, 325–338. 10.1107/S0907444910006694 PMC2852296 20382985

[bb67] Usón, I. & Sheldrick, G. M. (2018). *Acta Cryst.* D**74**, 106–116. 10.1107/S2059798317015121 PMC5947774 29533236

[bb68] Vagin, A. & Teplyakov, A. (2010). *Acta Cryst.* D**66**, 22–25. 10.1107/S0907444909042589 20057045

[bb69] Wang, J., Li, Y. & Modis, Y. (2014). *Acta Cryst.* D**70**, 1873–1883. 10.1107/S1399004714008943 PMC4089484 25004964

[bb70] Williams, C. J., Headd, J. J., Moriarty, N. W., Prisant, M. G., Videau, L. L., Deis, L. N., Verma, V., Keedy, D. A., Hintze, B. J., Chen, V. B., Jain, S., Lewis, S. M., Arendall, W. B. III, Snoeyink, J., Adams, P. D., Lovell, S. C., Richardson, J. S. & Richardson, D. C. (2018). *Protein Sci.* **27**, 293–315. 10.1002/pro.3330 PMC5734394 29067766

[bb71] Zimmermann, L., Stephens, A., Nam, S.-Z., Rau, D., Kübler, J., Lozajic, M., Gabler, F., Söding, J., Lupas, A. N. & Alva, V. (2018). *J. Mol. Biol.* **430**, 2237–2243. 10.1016/j.jmb.2017.12.007 29258817

