# Supplementary material for: A practical overview of molecular replacement: Clostridioides difficile PilA1, a difficult case study
Source: Acta Crystallogr D Struct Biol. 2020 Feb 26;76(Pt 3):261–71. doi: 10.1107/S2059798320000467 (PMC7057214; doi:10.1107/S2059798320000467)
Supplement: Supplementary file 1 [file d-76-00261-sup1.pdf]

STRUCTURAL
BIOLOGY

Volume 76 (2020)

Supporting information for article:

A practical overview of molecular replacement: *Clostridioides difficile* PilA1, a difficult case study

Adam D. Crawshaw, Arnaud Baslé and Paula S. Salgado

Table S1 PISA server interfaces

N#	Structure 1				Structure 2						
	Range	N_{at}	N_{res}	Surface Å^2	Range	Symmetry op.	N_{at}	N_{res}	Surface Å	Interface area Å^2	CSS
1	B	61	20	7240	A	-y+2, x+2, -z+1/2	61	17	7118	588.3	0
2	A	61	22	7118	B	-y+3/2, x-1/2, z+1/4	61	18	7240	515.8	0
3	C	59	15	7101	C	-y+3/2, x-1/2, z+1/4	57	22	7101	496.2	0
4	A	48	17	7118	A	-y+2, x+2, -z+1/2	49	17	7118	459.8	0
5	C	37	14	7101	B	x, y, z	40	14	7240	371.4	0
6	B	28	11	7240	A	x, y, z	33	11	7118	274.3	0
7	C	37	13	7101	B	-y+3/2, x-1/2, z+1/4	22	9	7240	227.6	0
8	C	11	5	7101	A	x, y, z	8	4	7118	78.0	0

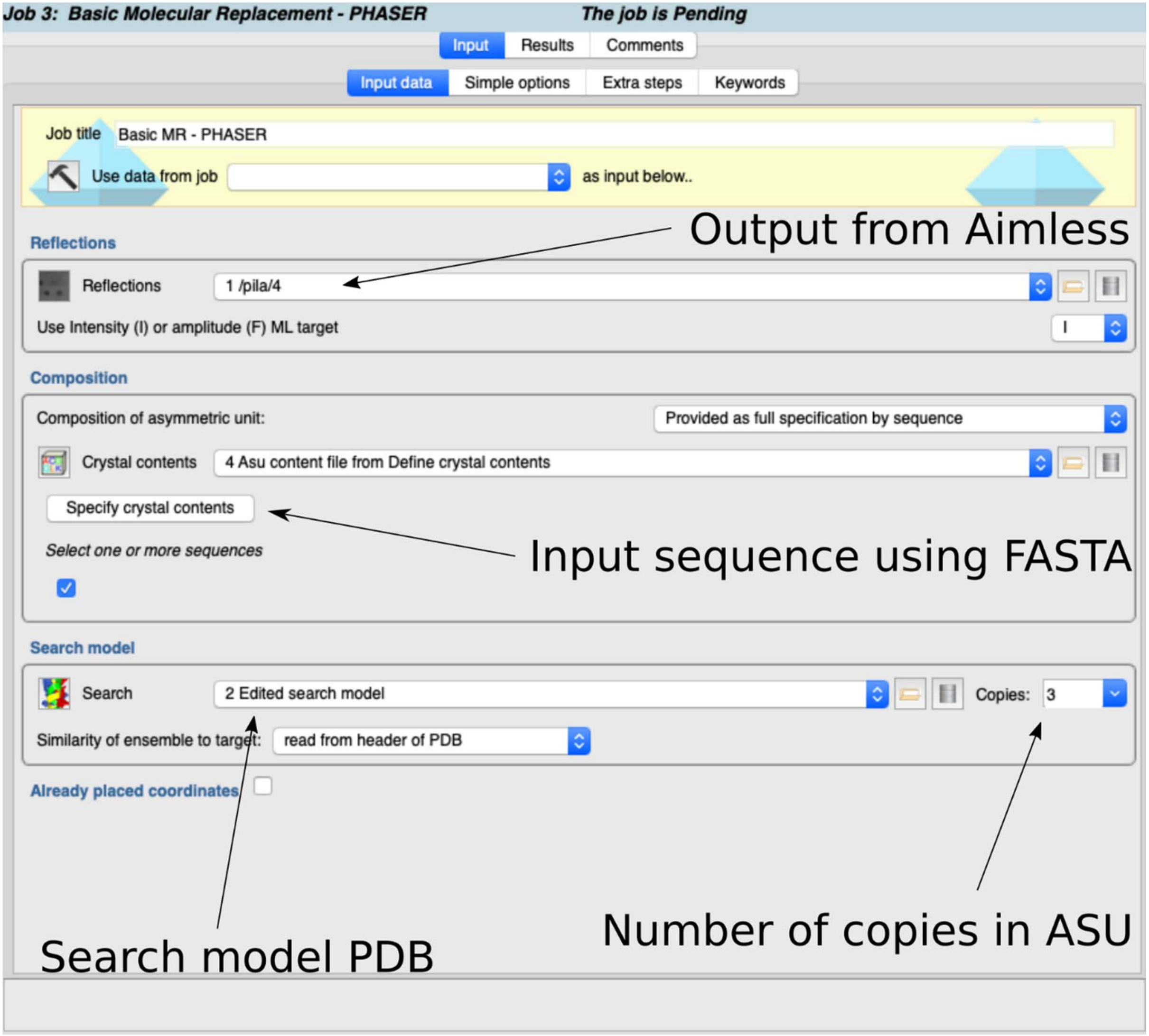

Figure S1 PHASER input form in the CCP4i2 suite. Basic molecular replacement requires scaled reflections, here selected from the AIMLESS task output, the crystal contents to be defined by input of a FASTA sequence of the target and a search model which has been selected from the output from SCULPTOR. Alignment information was included in the header of the output from SCULPTOR but can also be defined as sequence identity (0.1-0.9) or RMS value. Information must also be given regarding the expected number of molecules in the asymmetric unit.

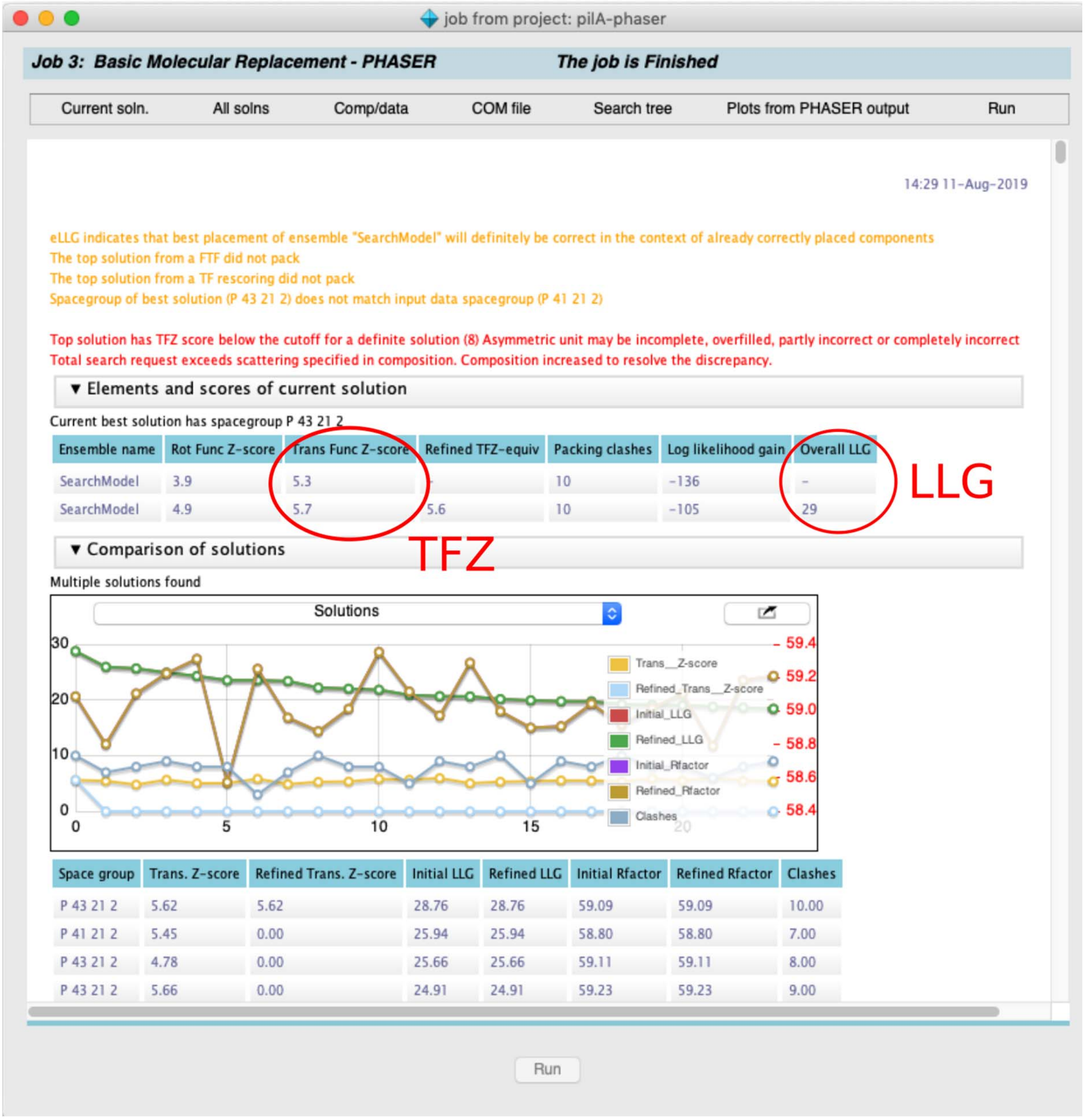

Figure S2 The output from PHASER viewed in CCP4i2 showing the statistics for the best result. The most important of these, the TFZ and LLG are circled in red. In the case of this process, the TFZ was 5.7 and LLG 29, indicating that a correct solution had not been found, as highlighted in the program output comments.
